# Crystal structure of *N*-(2-hy­droxy­eth­yl)-5-nitro­isophthalamic acid monohydrate

**DOI:** 10.1107/S160053681402337X

**Published:** 2014-10-29

**Authors:** Pei Zou, Hong-Yong Wang, Shi-Neng Luo, Ya-Ling Liu, Yong-Jia Shen

**Affiliations:** a Institute of Fine Chemicals, East China University of Science and Technology, Shanghai 200237, People’s Republic of China; b Jiangsu Institute of Nuclear Medicine, Wuxi 214063, People’s Republic of China

**Keywords:** crystal structure, isophthalamic acid, hydrogen bonding, C—H⋯O inter­actions, X-ray contrast media

## Abstract

In the title compound, C_10_H_10_N_2_O_6_·H_2_O, the carb­oxy­lic acid group and the nitro group are essentially coplanar with the benzene ring [maximum deviation = 0.0264 (9) Å], while the amide group is oriented at a dihedral angle of 9.22 (5)° with respect to the benzene ring. In the crystal, classical O—H⋯O and N—H⋯O hydrogen bonds and weak C—H⋯O inter­actions link the organic mol­ecules and water mol­ecules of crystallization into a three-dimensional supra­molecular architecture.

## Related literature   

The title compound is an inter­mediate for the preparation of iodinated X-ray contrast media, such as ioxitalamic acid and ioxilan, see: Prous *et al.* (1995 ▶); Sovak (1988 ▶); Stacul (2001 ▶). For a related structure, see: Liu *et al.* (2009 ▶).
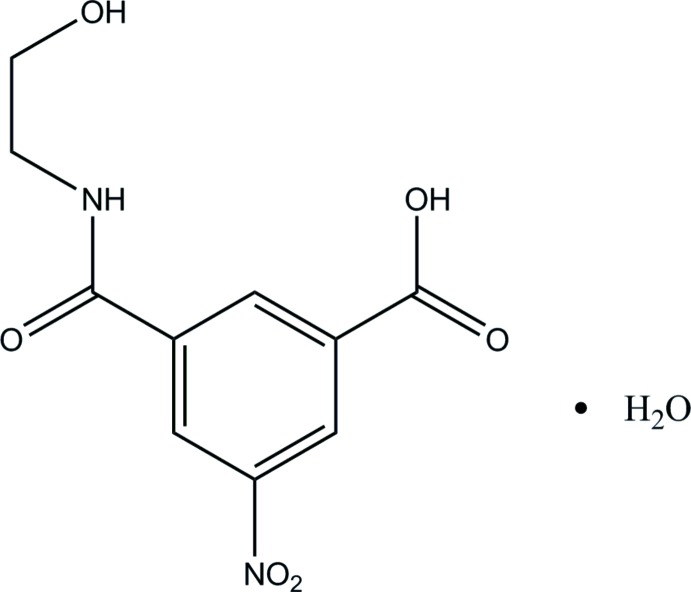


## Experimental   

### Crystal data  

C_10_H_10_N_2_O_6_·H_2_O
*M* *_r_* = 272.22
Triclinic, 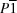

*a* = 6.449 (3) Å
*b* = 8.670 (5) Å
*c* = 11.051 (6) Å
α = 106.581 (8)°
β = 101.466 (9)°
γ = 93.692 (4)°
*V* = 575.6 (5) Å^3^
*Z* = 2
Mo *K*α radiation
μ = 0.14 mm^−1^
*T* = 173 K
0.44 × 0.31 × 0.06 mm

### Data collection  

Rigaku AFC10/Saturn724+ diffractometer
Absorption correction: multi-scan (*CrystalClear*; Rigaku/MSC, 2008 ▶) *T*_min_ = 0.93, *T*_max_ = 0.98
8661 measured reflections
3775 independent reflections
2826 reflections with *I* > 2σ(*I*)
*R*_int_ = 0.028

### Refinement  

*R*[*F* ^2^ > 2σ(*F* ^2^)] = 0.042
*wR*(*F* ^2^) = 0.110
*S* = 1.00
3775 reflections
192 parameters
H atoms treated by a mixture of independent and constrained refinement
Δρ_max_ = 0.41 e Å^−3^
Δρ_min_ = −0.21 e Å^−3^

### 

Data collection: *CrystalClear* (Rigaku/MSC, 2008 ▶); cell refinement: *CrystalClear*; data reduction: *CrystalClear*; program(s) used to solve structure: *SHELXTL* (Sheldrick, 2008 ▶); program(s) used to refine structure: *SHELXTL*; molecular graphics: *SHELXTL*; software used to prepare material for publication: *SHELXTL*.

## Supplementary Material

Crystal structure: contains datablock(s) I, global. DOI: 10.1107/S160053681402337X/xu5826sup1.cif

Structure factors: contains datablock(s) I. DOI: 10.1107/S160053681402337X/xu5826Isup2.hkl

Supporting information file. DOI: 10.1107/S160053681402337X/xu5826Isup3.cml

**Fig. 1.**
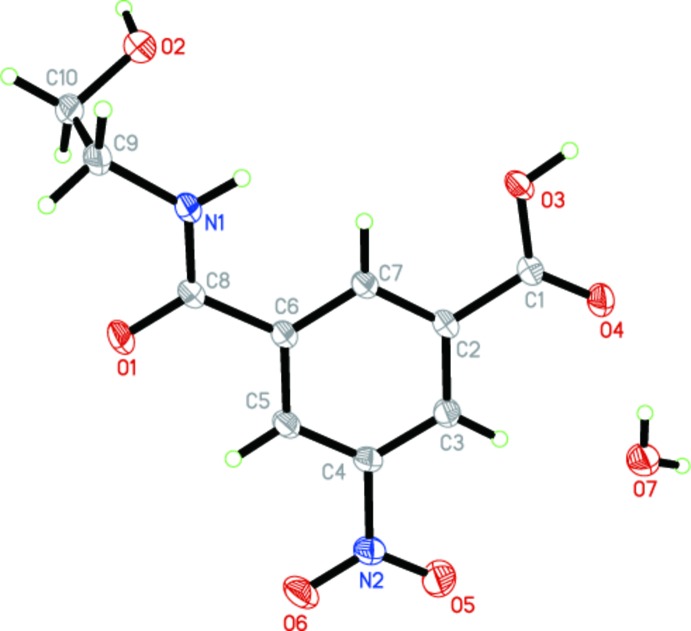
A view of the title compound with the atomic numbering scheme. Displacement ellipsoids were drawn at the 30% probability level.

. DOI: 10.1107/S160053681402337X/xu5826fig1.tif
A view of the title compound with the atomic numbering scheme. Displacement ellipsoids were drawn at the 30% probability level.

CCDC reference: 1030629

Additional supporting information:  crystallographic information; 3D view; checkCIF report

## Figures and Tables

**Table 1:** Hydrogen-bond geometry (, )

*D*H*A*	*D*H	H*A*	*D* *A*	*D*H*A*
N1H1*N*O2^i^	0.896(17)	2.103(16)	2.947(2)	156.6(13)
O2H2*O*O7^ii^	0.97(3)	1.78(3)	2.744(2)	169(2)
O3H3*O*O1^iii^	0.902(18)	1.677(18)	2.5601(19)	165.7(17)
O7H7*A*O4	0.81(2)	2.11(2)	2.887(2)	159.3(18)
O7H7*B*O2^iv^	0.87(2)	2.01(2)	2.853(2)	164(2)
C3H3O7	0.95	2.50	3.422(2)	163
C9H9*A*O7^v^	0.99	2.58	3.537(3)	164
C10H10*B*O3^i^	0.99	2.50	3.348(2)	143

Symmetry codes: (i) 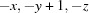; (ii) 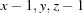; (iii) 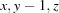; (iv) 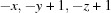; (v) 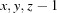.

## supplementary crystallographic information

### S1. Comment

The title compound has been used as an important inter­mediate for the preparation of iodinated X-ray contrast media, such as ioxitalamic acid and ioxilan, which are used clinically all over the world (Prous *et al.*, 1995; Sovak *et al.*, 1988; Stacul *et al.*, 2001). We report here the crystal structure of title compound.

The structure of the title compound is shown in Fig. 1 and hydrogen bond geometry is given in Table 1. The crystal data show that the bond lengths and angles are within expected ranges and agree well with the corresponding molecular dimensions reported for a similar compound (Liu *et al.*, 2009) . In the title compound, the carb­oxy­lic acid group and nitro group are approximately co-planar with the benzene ring [maximum deviation = 0.0264 (9) Å], while the amide moiety is oriented with respect to the benzene ring at 9.22 (5)°. In the crystal, classic O—H···O, N—H···O hydrogen bonds and weak C—H···O hydrogen inter­actions link organic molecules and crystalline water molecules into the three dimensional supra­molecular architecture.

### S2. Exprimental

Mono­methyl 5-nitro­benzene-1,3-di­hydrogencarboxyl­ate (900 mg, 4 mmol) was dissolved in methanol (5 ml), then ethano­lamine (610 mg, 10 mmol) was added and the mixture was refluxed for 16 h. Methanol was distilled off. The residue was dissolved in water and methanol (v/v 1:1), then acidified with 1 M hydro­chloric acid to pH = 3. The precipitate was filtered and washed with water. The crude product was recrystallized from ethanol/water. Single crystals were obtained by slow evaporation of an ethanol/water (v/v 7:1) solution.

### S3. Refinement

Hydroxyl H, water H and amino H atoms were located in a difference Fourier map and refined isotropically. Other H atoms were placed in calculated positions with C—H = 0.95–0.99 Å, and refined in riding mode, *U*_iso_(H) = 1.5*U*_eq_(C) for methyl H atoms and 1.2U_eq_(C) for the others.

### Crystal data

**Table:**

C_10_H_10_N_2_O_6_·H_2_O	*Z* = 2
*M**_r_* = 272.22	*F*(000) = 284
Triclinic, *P*1	*D*_x_ = 1.571 Mg m^−^^3^
Hall symbol: -P 1	Mo *K*α radiation, λ = 0.71073 Å
*a* = 6.449 (3) Å	Cell parameters from 2242 reflections
*b* = 8.670 (5) Å	θ = 2.5–31.5°
*c* = 11.051 (6) Å	µ = 0.14 mm^−^^1^
α = 106.581 (8)°	*T* = 173 K
β = 101.466 (9)°	Platelet, colorless
γ = 93.692 (4)°	0.44 × 0.31 × 0.06 mm
*V* = 575.6 (5) Å^3^	

### Data collection

**Table:**

Rigaku AFC10/Saturn724+ diffractometer	3775 independent reflections
Radiation source: Rotating Anode	2826 reflections with *I* > 2σ(*I*)
Graphite monochromator	*R*_int_ = 0.028
Detector resolution: 28.5714 pixels mm^-1^	θ_max_ = 31.5°, θ_min_ = 2.5°
phi and ω scans	*h* = −9→9
Absorption correction: multi-scan (*CrystalClear*; Rigaku/MSC, 2008)	*k* = −12→12
*T*_min_ = 0.93, *T*_max_ = 0.98	*l* = −16→16
8661 measured reflections	

### Refinement

**Table:**

Refinement on *F*^2^	Primary atom site location: structure-invariant direct methods
Least-squares matrix: full	Secondary atom site location: difference Fourier map
*R*[*F*^2^ > 2σ(*F*^2^)] = 0.042	Hydrogen site location: inferred from neighbouring sites
*wR*(*F*^2^) = 0.110	H atoms treated by a mixture of independent and constrained refinement
*S* = 1.00	*w* = 1/[σ^2^(*F*_o_^2^) + (0.0516*P*)^2^ + 0.0635*P*] where *P* = (*F*_o_^2^ + 2*F*_c_^2^)/3
3775 reflections	(Δ/σ)_max_ < 0.001
192 parameters	Δρ_max_ = 0.41 e Å^−^^3^
0 restraints	Δρ_min_ = −0.21 e Å^−^^3^

### Special details

**Table:**

Geometry. All e.s.d.'s (except the e.s.d. in the dihedral angle between two l.s. planes) are estimated using the full covariance matrix. The cell e.s.d.'s are taken into account individually in the estimation of e.s.d.'s in distances, angles and torsion angles; correlations between e.s.d.'s in cell parameters are only used when they are defined by crystal symmetry. An approximate (isotropic) treatment of cell e.s.d.'s is used for estimating e.s.d.'s involving l.s. planes.
Refinement. Refinement of *F*^2^ against ALL reflections. The weighted *R*-factor *wR* and goodness of fit *S* are based on *F*^2^, conventional *R*-factors *R* are based on *F*, with *F* set to zero for negative *F*^2^. The threshold expression of *F*^2^ > σ(*F*^2^) is used only for calculating *R*-factors(gt) *etc*. and is not relevant to the choice of reflections for refinement. *R*-factors based on *F*^2^ are statistically about twice as large as those based on *F*, and *R*- factors based on ALL data will be even larger.

### Fractional atomic coordinates and isotropic or equivalent isotropic displacement parameters (Å^2^)

**Table:**

	*x*	*y*	*z*	*U*_iso_*/*U*_eq_	
O1	0.13495 (17)	0.98481 (9)	0.27386 (8)	0.0329 (2)	
O2	−0.24748 (15)	0.59705 (10)	−0.05062 (8)	0.0295 (2)	
O3	0.18120 (14)	0.29530 (9)	0.34122 (8)	0.02551 (19)	
O4	0.26622 (14)	0.33382 (9)	0.55509 (8)	0.02415 (19)	
O5	0.39066 (19)	0.91355 (11)	0.83077 (8)	0.0419 (3)	
O6	0.36654 (17)	1.10655 (10)	0.74248 (9)	0.0336 (2)	
O7	0.33213 (16)	0.48483 (11)	0.83119 (10)	0.0321 (2)	
N1	0.13279 (17)	0.73307 (11)	0.14290 (9)	0.0232 (2)	
N2	0.35598 (17)	0.96255 (11)	0.73593 (9)	0.0246 (2)	
C1	0.23184 (17)	0.38719 (12)	0.46364 (10)	0.0183 (2)	
C2	0.24161 (17)	0.56423 (12)	0.47566 (10)	0.0175 (2)	
C3	0.29369 (17)	0.67808 (12)	0.59893 (10)	0.0190 (2)	
H3	0.3237	0.6453	0.6750	0.023*	
C4	0.30003 (17)	0.84077 (12)	0.60621 (10)	0.0192 (2)	
C5	0.25634 (17)	0.89481 (12)	0.49861 (10)	0.0197 (2)	
H5	0.2607	1.0074	0.5079	0.024*	
C6	0.20567 (17)	0.77954 (12)	0.37580 (10)	0.0183 (2)	
C7	0.19851 (17)	0.61473 (12)	0.36545 (10)	0.0184 (2)	
H7	0.1638	0.5361	0.2821	0.022*	
C8	0.15561 (19)	0.83987 (12)	0.25990 (10)	0.0208 (2)	
C9	0.0792 (2)	0.77886 (14)	0.02391 (11)	0.0269 (3)	
H9A	0.1418	0.7084	−0.0433	0.032*	
H9B	0.1419	0.8924	0.0411	0.032*	
C10	−0.1591 (2)	0.76327 (13)	−0.02582 (11)	0.0278 (3)	
H10A	−0.2227	0.8365	0.0393	0.033*	
H10B	−0.1913	0.7940	−0.1066	0.033*	
H1N	0.157 (2)	0.6305 (19)	0.1355 (14)	0.036 (4)*	
H2O	−0.397 (5)	0.569 (3)	−0.096 (3)	0.120 (10)*	
H3O	0.168 (3)	0.188 (2)	0.3314 (17)	0.053 (5)*	
H7A	0.317 (3)	0.421 (2)	0.759 (2)	0.060 (6)*	
H7B	0.302 (4)	0.440 (3)	0.888 (2)	0.089 (8)*	

### Atomic displacement parameters (Å^2^)

**Table:**

	*U*^11^	*U*^22^	*U*^33^	*U*^12^	*U*^13^	*U*^23^
O1	0.0600 (6)	0.0125 (4)	0.0251 (4)	0.0069 (4)	0.0059 (4)	0.0062 (3)
O2	0.0387 (5)	0.0219 (4)	0.0256 (4)	0.0048 (4)	0.0028 (4)	0.0064 (3)
O3	0.0422 (5)	0.0126 (3)	0.0208 (4)	0.0033 (3)	0.0063 (4)	0.0041 (3)
O4	0.0321 (5)	0.0200 (4)	0.0231 (4)	0.0051 (3)	0.0064 (3)	0.0104 (3)
O5	0.0755 (8)	0.0291 (5)	0.0179 (4)	0.0106 (5)	0.0042 (5)	0.0055 (3)
O6	0.0525 (6)	0.0160 (4)	0.0277 (5)	0.0007 (4)	0.0083 (4)	0.0010 (3)
O7	0.0448 (6)	0.0260 (5)	0.0239 (5)	0.0035 (4)	0.0052 (4)	0.0069 (4)
N1	0.0369 (6)	0.0134 (4)	0.0193 (4)	0.0037 (4)	0.0051 (4)	0.0055 (3)
N2	0.0317 (5)	0.0196 (4)	0.0195 (4)	0.0028 (4)	0.0048 (4)	0.0021 (3)
C1	0.0204 (5)	0.0153 (4)	0.0207 (5)	0.0035 (4)	0.0066 (4)	0.0063 (4)
C2	0.0187 (5)	0.0134 (4)	0.0211 (5)	0.0026 (4)	0.0058 (4)	0.0053 (4)
C3	0.0218 (5)	0.0172 (5)	0.0188 (5)	0.0035 (4)	0.0050 (4)	0.0060 (4)
C4	0.0221 (5)	0.0160 (5)	0.0171 (5)	0.0018 (4)	0.0039 (4)	0.0020 (4)
C5	0.0236 (5)	0.0133 (4)	0.0210 (5)	0.0014 (4)	0.0050 (4)	0.0036 (4)
C6	0.0220 (5)	0.0140 (4)	0.0188 (5)	0.0021 (4)	0.0043 (4)	0.0049 (4)
C7	0.0222 (5)	0.0139 (4)	0.0184 (5)	0.0022 (4)	0.0045 (4)	0.0041 (3)
C8	0.0273 (6)	0.0129 (4)	0.0207 (5)	0.0005 (4)	0.0039 (4)	0.0044 (4)
C9	0.0449 (7)	0.0175 (5)	0.0202 (5)	0.0030 (5)	0.0092 (5)	0.0077 (4)
C10	0.0466 (8)	0.0183 (5)	0.0189 (5)	0.0098 (5)	0.0064 (5)	0.0056 (4)

### Geometric parameters (Å, º)

**Table:**

O1—C8	1.2408 (14)	C2—C7	1.3921 (16)
O2—C10	1.4435 (16)	C2—C3	1.3967 (15)
O2—H2O	0.98 (3)	C3—C4	1.3871 (16)
O3—C1	1.3219 (14)	C3—H3	0.9500
O3—H3O	0.904 (19)	C4—C5	1.3837 (16)
O4—C1	1.2147 (14)	C5—C6	1.3983 (15)
O5—N2	1.2257 (14)	C5—H5	0.9500
O6—N2	1.2264 (14)	C6—C7	1.3974 (15)
O7—H7A	0.81 (2)	C6—C8	1.5027 (16)
O7—H7B	0.88 (3)	C7—H7	0.9500
N1—C8	1.3321 (15)	C9—C10	1.509 (2)
N1—C9	1.4632 (16)	C9—H9A	0.9900
N1—H1N	0.897 (16)	C9—H9B	0.9900
N2—C4	1.4770 (15)	C10—H10A	0.9900
C1—C2	1.4984 (16)	C10—H10B	0.9900
			
C10—O2—H2O	115.2 (15)	C6—C5—H5	120.8
C1—O3—H3O	113.6 (11)	C7—C6—C5	119.40 (10)
H7A—O7—H7B	113 (2)	C7—C6—C8	122.83 (9)
C8—N1—C9	122.19 (10)	C5—C6—C8	117.75 (10)
C8—N1—H1N	119.9 (10)	C2—C7—C6	120.85 (10)
C9—N1—H1N	117.8 (10)	C2—C7—H7	119.6
O5—N2—O6	123.86 (10)	C6—C7—H7	119.6
O5—N2—C4	117.95 (10)	O1—C8—N1	121.55 (10)
O6—N2—C4	118.19 (10)	O1—C8—C6	120.47 (10)
O4—C1—O3	123.79 (10)	N1—C8—C6	117.98 (10)
O4—C1—C2	124.30 (10)	N1—C9—C10	111.50 (10)
O3—C1—C2	111.91 (9)	N1—C9—H9A	109.3
C7—C2—C3	120.36 (10)	C10—C9—H9A	109.3
C7—C2—C1	120.47 (9)	N1—C9—H9B	109.3
C3—C2—C1	119.16 (9)	C10—C9—H9B	109.3
C4—C3—C2	117.51 (10)	H9A—C9—H9B	108.0
C4—C3—H3	121.2	O2—C10—C9	108.51 (9)
C2—C3—H3	121.2	O2—C10—H10A	110.0
C5—C4—C3	123.53 (10)	C9—C10—H10A	110.0
C5—C4—N2	118.37 (10)	O2—C10—H10B	110.0
C3—C4—N2	118.10 (10)	C9—C10—H10B	110.0
C4—C5—C6	118.34 (10)	H10A—C10—H10B	108.4
C4—C5—H5	120.8		
			
O4—C1—C2—C7	−179.61 (11)	C4—C5—C6—C7	0.66 (16)
O3—C1—C2—C7	0.41 (14)	C4—C5—C6—C8	179.17 (10)
O4—C1—C2—C3	0.13 (16)	C3—C2—C7—C6	−0.23 (16)
O3—C1—C2—C3	−179.85 (10)	C1—C2—C7—C6	179.51 (10)
C7—C2—C3—C4	0.08 (16)	C5—C6—C7—C2	−0.15 (16)
C1—C2—C3—C4	−179.66 (9)	C8—C6—C7—C2	−178.58 (10)
C2—C3—C4—C5	0.46 (17)	C9—N1—C8—O1	−0.92 (19)
C2—C3—C4—N2	−179.69 (10)	C9—N1—C8—C6	178.56 (10)
O5—N2—C4—C5	178.70 (11)	C7—C6—C8—O1	169.92 (11)
O6—N2—C4—C5	−1.81 (16)	C5—C6—C8—O1	−8.53 (17)
O5—N2—C4—C3	−1.16 (16)	C7—C6—C8—N1	−9.56 (17)
O6—N2—C4—C3	178.33 (10)	C5—C6—C8—N1	171.99 (11)
C3—C4—C5—C6	−0.84 (17)	C8—N1—C9—C10	−88.93 (13)
N2—C4—C5—C6	179.31 (10)	N1—C9—C10—O2	−59.16 (12)

### Hydrogen-bond geometry (Å, º)

**Table:**

*D*—H···*A*	*D*—H	H···*A*	*D*···*A*	*D*—H···*A*
N1—H1*N*···O2^i^	0.896 (17)	2.103 (16)	2.947 (2)	156.6 (13)
O2—H2*O*···O7^ii^	0.97 (3)	1.78 (3)	2.744 (2)	169 (2)
O3—H3*O*···O1^iii^	0.902 (18)	1.677 (18)	2.5601 (19)	165.7 (17)
O7—H7*A*···O4	0.81 (2)	2.11 (2)	2.887 (2)	159.3 (18)
O7—H7*B*···O2^iv^	0.87 (2)	2.01 (2)	2.853 (2)	164 (2)
C3—H3···O7	0.95	2.50	3.422 (2)	163
C9—H9*A*···O7^v^	0.99	2.58	3.537 (3)	164
C10—H10*B*···O3^i^	0.99	2.50	3.348 (2)	143

Symmetry codes: (i) −*x*, −*y*+1, −*z*; (ii) *x*−1, *y*, *z*−1; (iii) *x*, *y*−1, *z*; (iv) −*x*, −*y*+1, −*z*+1; (v) *x*, *y*, *z*−1.
