# Supplementary figures and images for: Crystal structure of N-(2-hy­droxy­eth­yl)-5-nitro­isophthalamic acid monohydrate
Source: Acta Crystallogr Sect E Struct Rep Online. 2014 Oct 29;70(Pt 11):o1205–6. doi: 10.1107/S160053681402337X (PMC4257245; doi:10.1107/S160053681402337X)

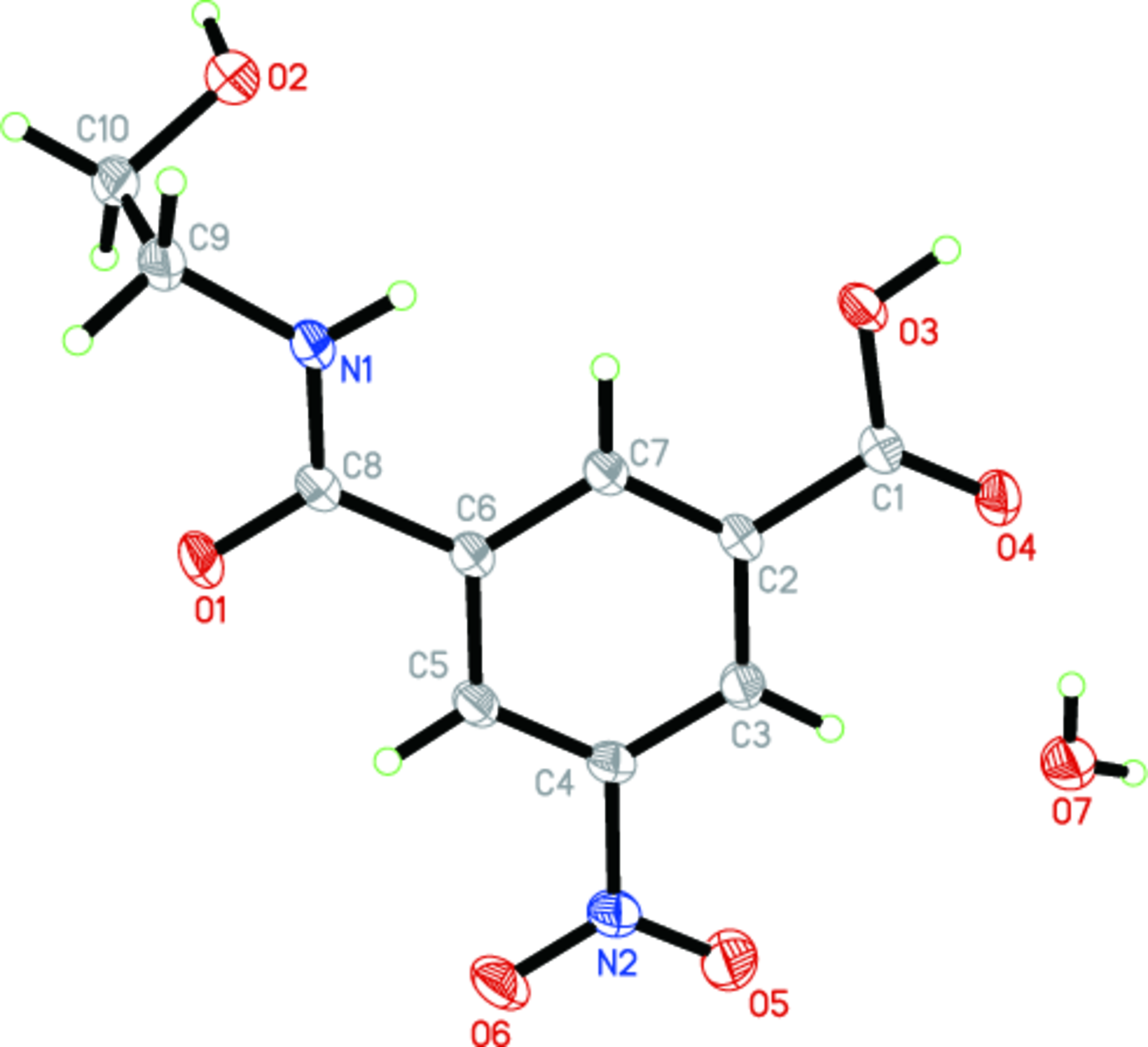

Supplement: Supplementary file 4 [file e-70-o1205-fig1.tif]
